# Immune checkpoint inhibitor–based combination therapy for platinum-resistant or platinum-refractory recurrent ovarian cancer: a systematic review and meta-analysis of prospective trials

**DOI:** 10.3389/fonc.2026.1837967

**Published:** 2026-08-26

**Authors:** Jing Xu, Lin Zhang, Wen-Ying Yang, Xiao-Jing Cai, Xiong Xiao

**Affiliations:** 1 Department of Oncology, The Second School of Clinical Medicine, Guizhou University of Traditional Chinese Medicine, Guiyang City, Guizhou, China; 2 Department of Oncology, The Second Affiliated Hospital of Guizhou University of Traditional Chinese Medicine, Guiyang City, Guizhou, China

**Keywords:** immune checkpoint inhibitor, meta-analysis, objective response rate, pembrolizumab, platinum-resistant ovarian cancer, systematic review

## Abstract

**Background:**

The clinical value of immune checkpoint inhibitor (ICI)-based combinations in platinum-resistant or platinum-refractory recurrent ovarian cancer remains regimen-dependent and uncertain. We updated the evidence base and separated nonrandomized response evidence from randomized comparative evidence.

**Methods:**

PubMed/MEDLINE, Embase, Web of Science, and the Cochrane Central Register of Controlled Trials (CENTRAL) were searched from inception to 23 July 2026, supplemented by reference and citation searching. Eligible reports were peer-reviewed prospective phase II or III trials of an ICI-containing combination in adults with platinum-resistant or platinum-refractory recurrent epithelial ovarian, fallopian tube, or primary peritoneal cancer. Front-line, maintenance-only, ICI-monotherapy, retrospective, phase I-only, protocol-only, and mixed platinum-status cohorts without extractable resistant/refractory data were excluded. Objective response rate (ORR) and disease control rate (DCR) from compatible nonrandomized cohorts were synthesized as proportions using a logit random-effects model with Paule–Mandel variance estimation and Hartung–Knapp confidence intervals. Randomized hazard ratios were synthesized descriptively and were not pooled.

**Results:**

Twenty-one eligible prospective reports were included, comprising 14 nonrandomized reports and 7 randomized trials. Of the 14 nonrandomized reports, 13 provided compatible confirmed response data for the pooled ORR analysis, yielding 112 objective responses among 490 participants and a pooled ORR of 21.9% (95% CI, 13.1%–34.3%; I² = 70.7%). The remaining nonrandomized report was retained for narrative synthesis only because it reported an unconfirmed response. Ten nonrandomized reports contributed 211 disease-control events among 354 participants, yielding a pooled DCR of 59.9% (95% CI, 46.4%–72.1%; I² = 71.7%). The seven randomized trials were summarized separately and were not pooled with the nonrandomized reports. KEYNOTE-B96 demonstrated improved progression-free survival (HR, 0.70; 95% CI, 0.58–0.84) and overall survival (HR, 0.82; 95% CI, 0.69–0.97), whereas JAVELIN Ovarian 200, EORTC 1508, NRG-GY023, and AGO-OVAR 2.29 did not meet their primary efficacy objectives.

**Conclusions:**

ICI-based combinations show measurable but highly heterogeneous activity in platinum-resistant/refractory ovarian cancer. The evidence does not support a uniform class effect: a survival benefit is established for pembrolizumab plus weekly paclitaxel in KEYNOTE-B96, whereas several other randomized strategies were negative. Future trials should prioritize regimen-specific validation, biomarker enrichment, and harmonized toxicity reporting.

## 1 Introduction

Platinum-resistant or platinum-refractory recurrent ovarian cancer remains a difficult setting in gynecologic oncology. Disease that progresses during platinum treatment or recurs within 6 months is characterized by limited sensitivity to cytotoxic therapy, short response duration, cumulative treatment toxicity, and fewer active options (1, 2). Although weekly paclitaxel, pegylated liposomal doxorubicin, gemcitabine, topotecan, and targeted agents are used, no standard provides durable disease control for most patients. This context is distinct from platinum-sensitive relapse, first-line therapy, and maintenance treatment and therefore requires evidence evaluated within a clearly defined resistant or refractory population. Immune checkpoint inhibitors (ICIs) targeting programmed cell death protein 1 or programmed death-ligand 1 have transformed several solid tumors but have shown modest single-agent activity in unselected ovarian cancer (3, 4). Ovarian tumors may exhibit heterogeneous PD-L1 expression, variable tumor mutational burden, an immunosuppressive microenvironment, and impaired T-cell infiltration (5). Combination approaches therefore seek to enhance antigen release, innate immune signaling, vascular normalization, or T-cell activation through chemotherapy, antiangiogenic agents, poly(ADP-ribose) polymerase inhibitors, antibody–drug conjugates, vaccines, epigenetic therapies, or dual-checkpoint blockade (6). These approaches involve partners, toxicities, and selection strategies; ICI-containing regimens should therefore not be assumed to have a common therapeutic effect.

Clinical evidence has reflected this heterogeneity. JAVELIN Ovarian 200 did not show a statistically significant improvement in progression-free survival (PFS) or overall survival (OS) with avelumab plus pegylated liposomal doxorubicin compared with chemotherapy alone (7, 8). By contrast, KEYNOTE-B96 subsequently demonstrated PFS and OS benefits with pembrolizumab plus weekly paclitaxel in the intention-to-treat population. Between these phase III trials, multiple prospective phase II studies evaluated ICIs in combination with poly(ADP-ribose) polymerase (PARP) inhibition, antiangiogenic therapy, cytotoxic chemotherapy, vaccination, epigenetic modification, or antibody–drug conjugates (9). Most were small, nonrandomized, and designed to identify activity signals rather than comparative efficacy; their response estimates varied substantially (10).

These differences create two methodological problems. First, a response proportion from a single-arm cohort cannot be interpreted as a risk ratio or combined directly with a randomized treatment effect (11, 12). Second, broad eligibility criteria may mix front-line, maintenance, platinum-sensitive, and platinum-resistant settings, obscuring the population to which an estimate applies (13, 14). We therefore updated the literature search and conducted a design-stratified systematic review restricted to recurrent platinum-resistant or platinum-refractory ovarian, fallopian tube, or primary peritoneal cancer. We aimed to synthesize objective response and disease control from compatible prospective nonrandomized cohorts, summarize randomized PFS and OS effects without inappropriate pooling, evaluate regimen-specific safety, and identify priorities for future trials.

## 2 Methods

### 2.1 Reporting framework and search strategy

The review was reported in accordance with Preferred Reporting Items for Systematic Reviews and Meta-Analyses (PRISMA) 2020 (15). PubMed/MEDLINE, Embase, Web of Science, and the Cochrane Central Register of Controlled Trials (CENTRAL) were searched from inception to 23 July 2026 without language restrictions. The strategy combined database-specific controlled vocabulary and free-text terms for ovarian, fallopian tube, and primary peritoneal cancer; platinum resistance or refractoriness; immune checkpoint inhibitors and individual agents; combination treatment; and prospective phase II or III trials. Reference lists of eligible reports and relevant reviews were examined, and cited-by records of eligible trials were screened using the same Population, Intervention, Comparator, Outcomes, and Study design (PICOS) criteria. The database search framework, search date, screening counts, and report-level exclusion log are provided in **Supplementary Table 1**. Detailed study characteristics, extracted efficacy data, and design-specific risk-of-bias assessments are provided in **Supplementary Tables 2**–**S4**, respectively.

### 2.2 Eligibility criteria

Population: adults with recurrent epithelial ovarian, fallopian tube, or primary peritoneal cancer explicitly defined as platinum-resistant (recurrence/progression within 6 months after platinum) or platinum-refractory; mixed populations were eligible only when resistant/refractory data were separately extractable. Intervention: an active ICI-containing combination. Comparator: any concurrent control for randomized trials; none required for prospective nonrandomized phase II cohorts. Outcomes: ORR, DCR, PFS, OS, and treatment-emergent or treatment-related adverse events. Study design: peer-reviewed prospective phase II or III trials; a phase I/II report was eligible only when a prespecified phase II efficacy cohort was reported. Front-line trials, maintenance-only trials after response, ICI monotherapy, phase I-only studies, retrospective series, case reports, protocols, reviews, and overlapping/secondary reports were excluded.

### 2.3 Study selection and data extraction

Two reviewers independently screened records and extracted study design, population, regimen, analysis denominator, response counts, time-to-event estimates, and safety outcomes; discrepancies were resolved by consensus. Counts were taken from the analysis population specified by each primary publication. Confirmed complete plus partial responses defined ORR. DCR required compatible counts for complete response, partial response, and stable disease; a trial reporting only an unconfirmed response was retained for narrative synthesis but excluded from the quantitative ORR analysis. When multiple ICI-containing cohorts occurred within one nonrandomized report, independent cohorts were combined once at report level.

### 2.4 Outcome synthesis and handling of study design

Nonrandomized prospective cohorts contributed study-level response proportions, not risk ratios. Experimental arms from randomized trials were not mixed into the primary proportion meta-analysis. Randomized trials contributed comparative estimates exactly as published. Because comparators, regimens, primary endpoints, analysis populations, and even reported confidence levels differed, randomized PFS and OS hazard ratios were displayed but not pooled. Safety was synthesized narratively because trials variably reported adverse events, treatment-emergent adverse events, treatment-related adverse events, immune-mediated events, and different observation windows.

### 2.5 Risk-of-bias assessment

Nonrandomized prospective trials were assessed with the Methodological Index for Non-Randomized Studies (MINORS); single-arm studies were scored against the eight noncomparative items (maximum 16) (16). Randomized trials were assessed with Cochrane RoB 2 across bias arising from the randomization process, deviations from intended interventions, missing outcome data, measurement of the outcome, and selection of the reported result (17). Judgments and supporting notes are reported in **Supplementary Table 4**.

### 2.6 Statistical analysis

For compatible nonrandomized cohorts, proportions were transformed to logits and synthesized with a random-effects model. Between-study variance was estimated by the Paule–Mandel iterative method (18). The pooled 95% confidence interval used the Hartung–Knapp adjustment (19). A 0.5 continuity correction was applied only to a boundary cell (zero or all events). Individual-study intervals were exact Clopper–Pearson intervals (20). Heterogeneity was summarized with Cochran’s Q, τ², and I². Prespecified exploratory subgroup analyses were conducted by combination class when at least two cohorts were available. Robustness was examined by leave-one-out analysis. Funnel-plot asymmetry testing was not used because proportion meta-analysis, small subgroup sizes, and marked clinical heterogeneity make such tests difficult to interpret. Analyses were performed in Python 3 using statsmodels 0.14.6 and SciPy 1.18.0; the extracted aggregate counts are reproduced in **Supplementary Table 3**.

## 3 Results

### 3.1 Study selection

**Figure 1:**
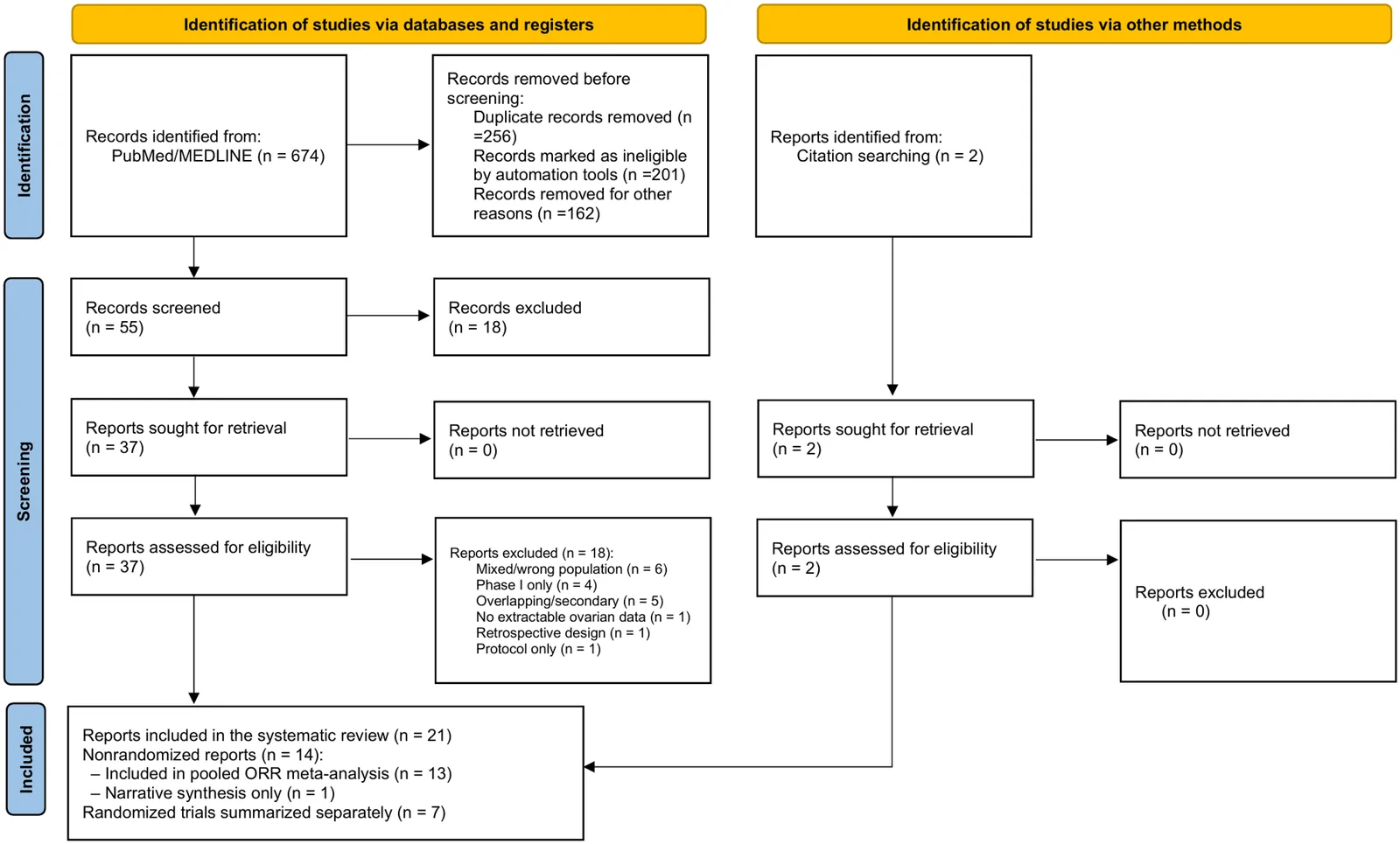
PRISMA 2020 flow diagram for the updated search and study selection. The database searches yielded 674 records, and reference and citation searching identified two additional reports. Twenty-one prospective trial reports met the revised platinum-resistant/refractory eligibility criteria.

The database searches identified 674 records, and reference and citation searching identified two additional reports. Before screening, 619 database records were removed: 256 duplicates, 201 records excluded by prespecified record-level filters, and 162 records removed for other prespecified reasons. The remaining 55 records underwent title and abstract screening, and 18 were excluded. Thirty-seven reports were sought and retrieved for full-text assessment. Both reports identified through reference and citation searching were also retrieved, resulting in 39 full-text reports assessed for eligibility. Eighteen reports were excluded because of a mixed or incorrect study population (n = 6), phase I-only design (n = 4), overlapping or secondary publication (n = 5), absence of extractable ovarian-specific data (n = 1), retrospective design (n = 1), or protocol-only publication (n = 1). Twenty-one reports met the eligibility criteria and were included in the systematic review. Twenty-one prospective primary reports met the eligibility criteria, comprising 14 nonrandomized reports and 7 randomized trials. Thirteen nonrandomized reports provided compatible confirmed response data and contributed to the pooled ORR meta-analysis. The remaining nonrandomized report, Zamarin et al. (2020), was a prospective, single-arm phase II study and was retained for narrative synthesis only because its sole reported partial response was unconfirmed. The seven randomized trials contributed separate published comparative estimates and were not pooled with the nonrandomized reports (**Figure 1**).

### 3.2 Study characteristics

The 21 reports were published from 2019 through 2026 and evaluated chemotherapy, PARP inhibition, antiangiogenic agents, antibody–drug conjugates, vaccination, epigenetic therapy, or dual-checkpoint blockade combined with an ICI (1, 8, 9, 21–38). Sample sizes ranged from 20 in the two IPROC substudies to 643 in KEYNOTE-B96. The evidence base included small signal-seeking phase II cohorts, randomized phase II selection studies, and three phase III trials (**Table 1**; **Supplementary Table 2**).

**Table 1:** Characteristics and synthesis roles of the 21 included prospective reports.

Report	Design	Eligible population	ICI combination	Analyzed N	Synthesis role
Konstantinopoulos 2019 (TOPACIO)	Phase I/II, single-arm	Recurrent platinum-resistant ovarian carcinoma	Pembrolizumab + niraparib	62 treated; 60 efficacy-evaluable	Primary ORR/DCR meta-analysis
Lee 2020	Phase II, single-arm	Platinum-resistant ovarian cancer	Pembrolizumab + PLD	26 treated; 23 response-evaluable	Primary ORR meta-analysis
Zamarin 2020	Phase II, single-arm	Advanced platinum-resistant ovarian cancer	Durvalumab + TPIV200 FRα vaccine	27	Narrative synthesis; response unconfirmed
Liao 2021	Phase II, single-arm	Recurrent platinum-resistant ovarian/FT/PP cancer	Pembrolizumab + low-dose carboplatin	29	Primary ORR/DCR meta-analysis
Walsh 2021	Phase II, single-arm	Platinum-resistant ovarian cancer	Pembrolizumab + cisplatin + gemcitabine	21 treated; 18 response-evaluable	Primary ORR/DCR meta-analysis
Pujade-Lauraine 2021 (JAVELIN Ovarian 200)	Phase III, open-label randomized	Platinum-resistant/refractory ovarian cancer	Avelumab + PLD vs avelumab vs PLD	566 randomized; 188 combination arm	Randomized comparative synthesis
Xia 2022	Phase II, single-arm ovarian cohort	Platinum-resistant recurrent ovarian cancer	Camrelizumab + famitinib	37	Primary ORR/DCR meta-analysis
Randall 2023 (MOONSTONE)	Phase II, single-arm	Recurrent platinum-resistant ovarian cancer	Dostarlimab + niraparib	41	Primary ORR/DCR meta-analysis
Kim 2023 (KGOG 3045-HRR)	Phase II, randomized noncomparative	HRR-mutated platinum-resistant ovarian cancer	Olaparib + durvalumab vs olaparib + cediranib	30; 14 in ICI arm	Randomized evidence; not proportion-pooled
Hinchcliff 2024	Phase II, randomized open-label	Recurrent platinum-resistant ovarian cancer	Concurrent vs sequential durvalumab + tremelimumab	61 (23 concurrent; 38 sequential)	Randomized comparative synthesis
Kim 2024 (KGOG 3045-non-HRR)	Phase II umbrella trial	HRR-nonmutated platinum-resistant ovarian cancer	Durvalumab ± tremelimumab + chemotherapy	58 across four ICI-containing arms	Primary ORR meta-analysis
Liu 2024 (OPAL)	Phase II, single-arm cohort	Pretreated platinum-resistant ovarian cancer	Dostarlimab + niraparib + bevacizumab	41	Primary ORR/DCR meta-analysis
Banerjee 2025 (EORTC 1508)	Phase II, randomized multicenter	Recurrent platinum-resistant ovarian cancer	Bevacizumab ± atezolizumab ± aspirin	96 in three reported active cohorts	Randomized comparative synthesis
Lee 2025 (NRG-GY023)	Phase II, randomized selection design	Platinum-resistant ovarian cancer after bevacizumab	Durvalumab + cediranib ± olaparib vs SOC/other	153; 86 in ICI-containing arms	Randomized comparative synthesis
Su 2025	Phase II, single-arm	Platinum-resistant recurrent ovarian cancer	Toripalimab + low-dose lenvatinib	33	Primary ORR/DCR meta-analysis
Matulonis 2025	Phase II, single-arm	FRα-positive platinum-resistant ovarian cancer	Pembrolizumab + mirvetuximab soravtansine	56 treated; 55 response-evaluable	Primary ORR meta-analysis
Harter 2026 (AGO-OVAR 2.29)	Phase III, double-blind randomized	Recurrent ovarian cancer ineligible for platinum	Atezolizumab vs placebo + bevacizumab + chemotherapy	574	Randomized comparative synthesis
Landon 2026	Phase II, nonrandomized	Platinum-resistant epithelial ovarian cancer	Pembrolizumab + oral azacitidine	34	Primary ORR/DCR meta-analysis
Wenham 2026	Phase II, single-arm multicenter	Platinum-resistant epithelial ovarian cancer	Pembrolizumab + weekly paclitaxel	42 enrolled; 41 ITT	Primary ORR/DCR meta-analysis
MacKay 2026 (IPROC A/B)	Phase II, nonrandomized platform	Platinum-resistant high-grade serous ovarian cancer	Durvalumab + mecbotamab or ozuriftamab vedotin	20 (10 per substudy)	Primary ORR/DCR meta-analysis
Colombo 2026 (KEYNOTE-B96)	Phase III, double-blind randomized	Platinum-resistant recurrent ovarian cancer	Pembrolizumab vs placebo + weekly paclitaxel	643 randomized (322 vs 321)	Randomized comparative synthesis

ADC, antibody–drug conjugate; FRα, folate receptor alpha; FT, fallopian tube; HRR, homologous recombination repair; ICI, immune checkpoint inhibitor; ITT, intention-to-treat; PARP, poly(ADP-ribose) polymerase; PLD, pegylated liposomal doxorubicin; PP, primary peritoneal; SOC, standard of care.

### 3.3 Risk of bias

The two double-blind phase III trials were judged at low overall risk of bias. Open-label randomized trials and randomized selection designs were generally rated as having some concerns, principally because treatment assignment was known and several studies were not designed for a definitive between-arm comparison. MINORS scores for nonrandomized reports ranged from 12 to 13 of 16; the dominant limitation was absence of a concurrent comparator, followed by small cohorts, early stopping, or incomplete response-evaluable populations. No nonrandomized study was interpreted as providing comparative causal evidence (**Supplementary Table 4**).

### 3.4 Objective response rate

**Figure 2:**
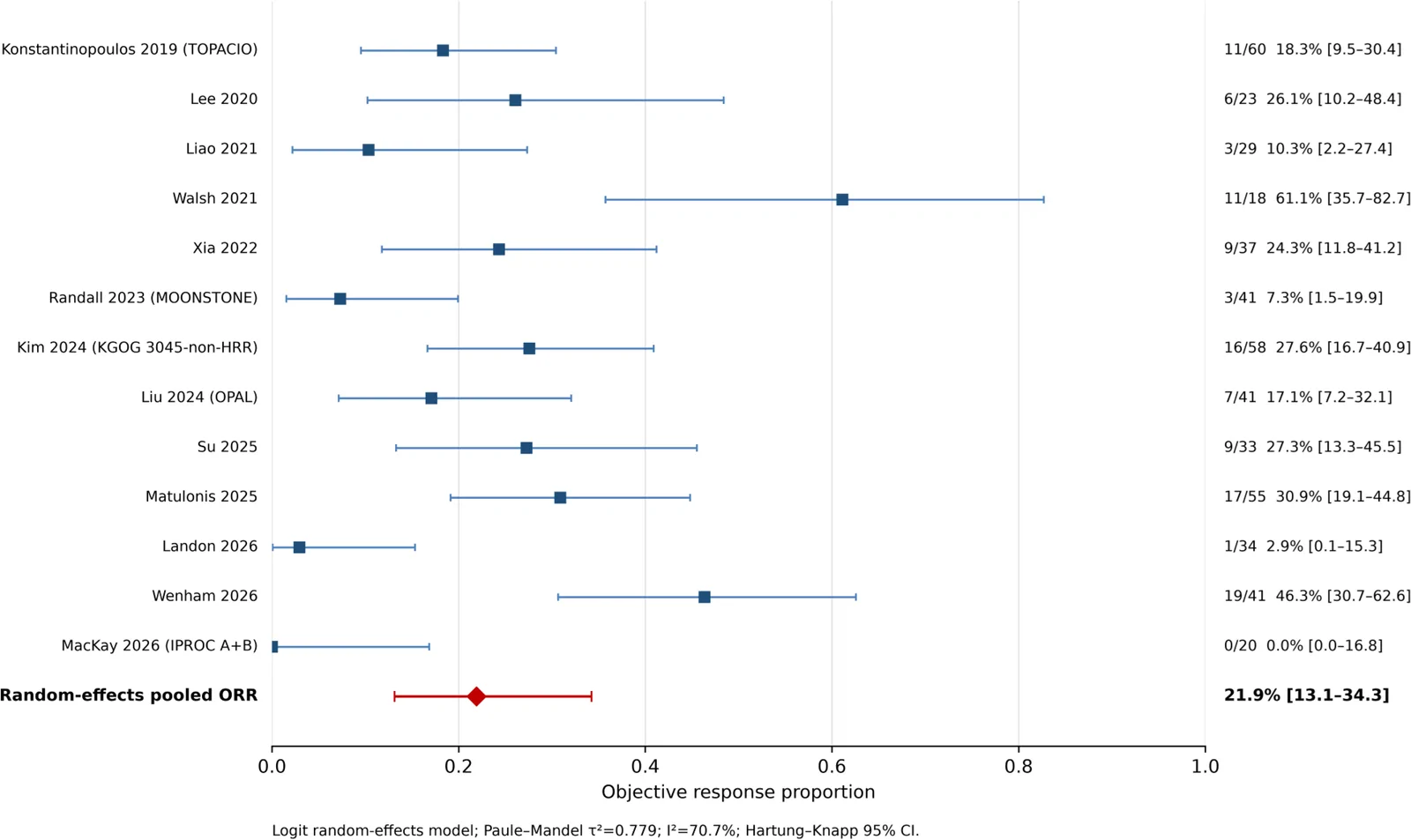
Forest plot of confirmed objective response proportions in 13 prospective nonrandomized cohorts. Squares show study proportions with exact Clopper–Pearson 95% confidence intervals. The diamond shows the logit random-effects pooled estimate with a Hartung–Knapp 95% confidence interval.

Thirteen nonrandomized prospective reports provided compatible confirmed response counts. Across 490 participants, 112 objective responses occurred. The pooled ORR was 21.9% (95% CI 13.1%–34.3%), with substantial heterogeneity (I²=70.7%; τ²=0.779; Q = 40.96, df=12) (**Figure 2**). This estimate quantifies average single-arm activity; it does not demonstrate superiority over standard treatment.

### 3.5 Disease control rate

Ten nonrandomized reports used compatible disease-control definitions and contributed 211 events among 354 participants. The pooled DCR was 59.9% (95% CI 46.4%–72.1%), with substantial heterogeneity (I²=71.7%; τ²=0.429). Studies using a durable-clinical-benefit definition rather than conventional complete response plus partial response plus stable disease were not combined (**Table 2**).

**Table 2:** Quantitative synthesis and randomized evidence.

Evidence stream	Reports/participants	Estimate	Heterogeneity/interpretation
Nonrandomized ORR	13/490	21.9% (95% CI 13.1–34.3)	I²=70.7%; single-arm activity only
Nonrandomized DCR	10/354	59.9% (95% CI 46.4–72.1)	I²=71.7%; compatible definitions only
Randomized evidence	7 randomized trials	Trial-specific response or survival endpoints	Not pooled because designs, regimens, comparators, endpoints, and CI levels differed

### 3.6 Randomized comparative evidence

**Figure 3:**
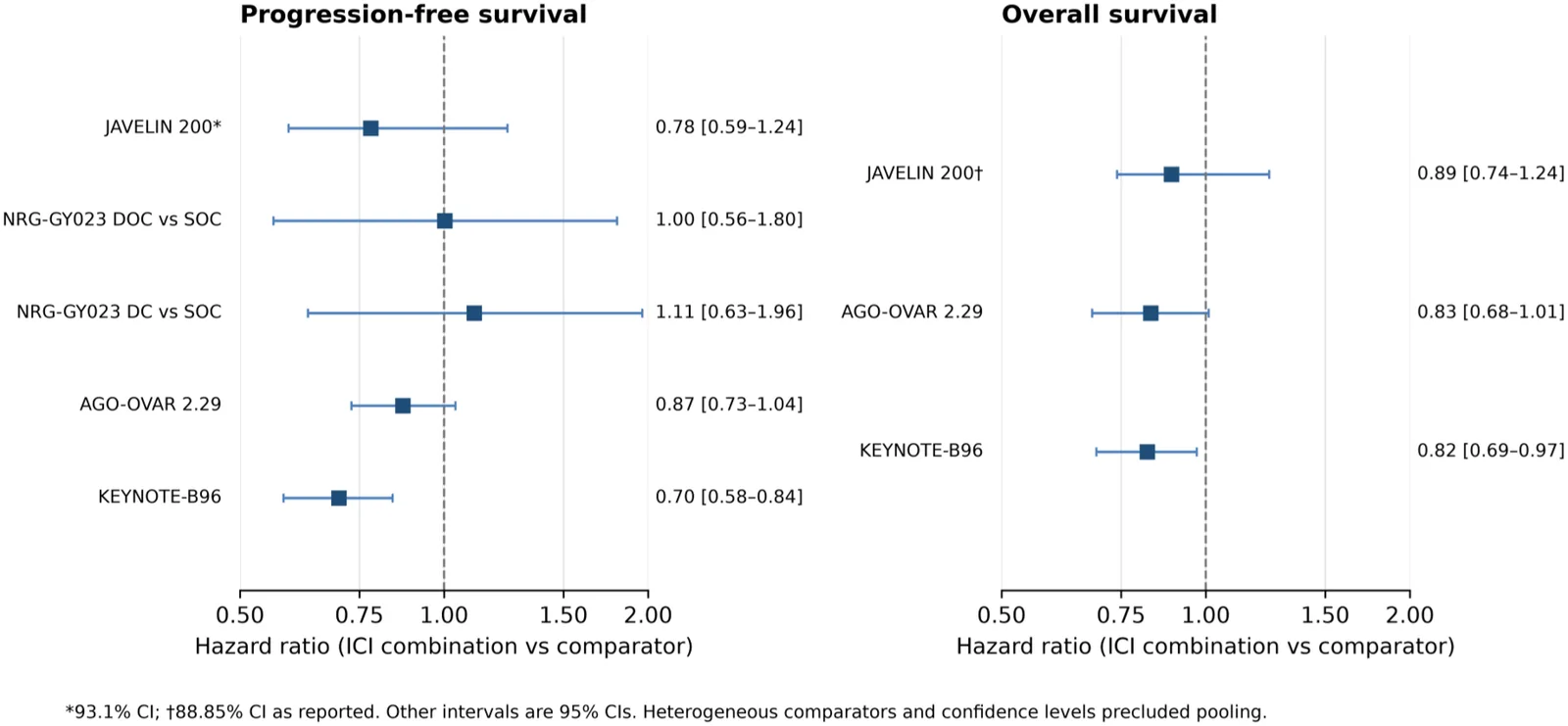
Published randomized comparative hazard ratios for progression-free survival (PFS) and overall survival (OS). No pooled estimate is shown because regimens, comparators, endpoints, and confidence levels differed. JAVELIN Ovarian 200 reported nonstandard confidence levels as marked. DC, durvalumab plus cediranib; DOC, durvalumab plus olaparib plus cediranib; SOC, standard of care.

The randomized trials did not support a uniform ICI-combination effect. The KGOG 3045-HRR study was randomized but explicitly noncomparative; its olaparib–durvalumab arm achieved 6 responses among 14 participants and was not used to estimate a treatment contrast (28). Concurrent versus sequential durvalumab–tremelimumab produced similar median immune-related PFS (1.87 vs 1.84 months; P = 0.402) (29). In JAVELIN Ovarian 200, avelumab plus PLD did not significantly improve the coprimary endpoints (PFS HR 0.78, reported 93.1% CI 0.59–1.24; OS HR 0.89, reported 88.85% CI 0.74–1.24) (8). EORTC 1508 did not meet its PFS-at-6-month primary objective in any reported arm (32). In NRG-GY023, the two durvalumab-containing arms crossed futility boundaries (PFS HR 1.108, 95% CI 0.63–1.96, for durvalumab plus cediranib; HR 1.003, 95% CI 0.56–1.80, for durvalumab plus olaparib plus cediranib, each versus standard of care) (33). AGO-OVAR 2.29 did not significantly improve PFS (HR 0.87, 95% CI 0.73–1.04) or OS (HR 0.83, 95% CI 0.68–1.01) (36). In contrast, KEYNOTE-B96 improved PFS (HR 0.70, 95% CI 0.58–0.84) and OS (HR 0.82, 95% CI 0.69–0.97) in the intention-to-treat population (9). Available comparative HRs are displayed without a pooled diamond in **Figure 3**.

### 3.7 Safety

Toxicity could not be summarized as a common risk ratio because reporting constructs and denominators differed. In KEYNOTE-B96, grade 3 or worse treatment-related adverse events occurred in 217/320 (67.8%) pembrolizumab-treated participants and 176/318 (55.3%) controls (9). In AGO-OVAR 2.29, grade ≥3 adverse events occurred in 72% and 69%, respectively (36). In the mirvetuximab–pembrolizumab cohort, pneumonitis occurred in 14/56 (25.0%) participants and was grade ≥3 in 2/56 (3.6%) (35). In the two IPROC substudies, grade 3 treatment-related adverse events occurred in 6/10 and 1/10 participants, respectively (1). These examples illustrate regimen-specific toxicity and should not be interpreted as a pooled class estimate.

### 3.8 Sensitivity and exploratory subgroup analyses

**Figure 4:**
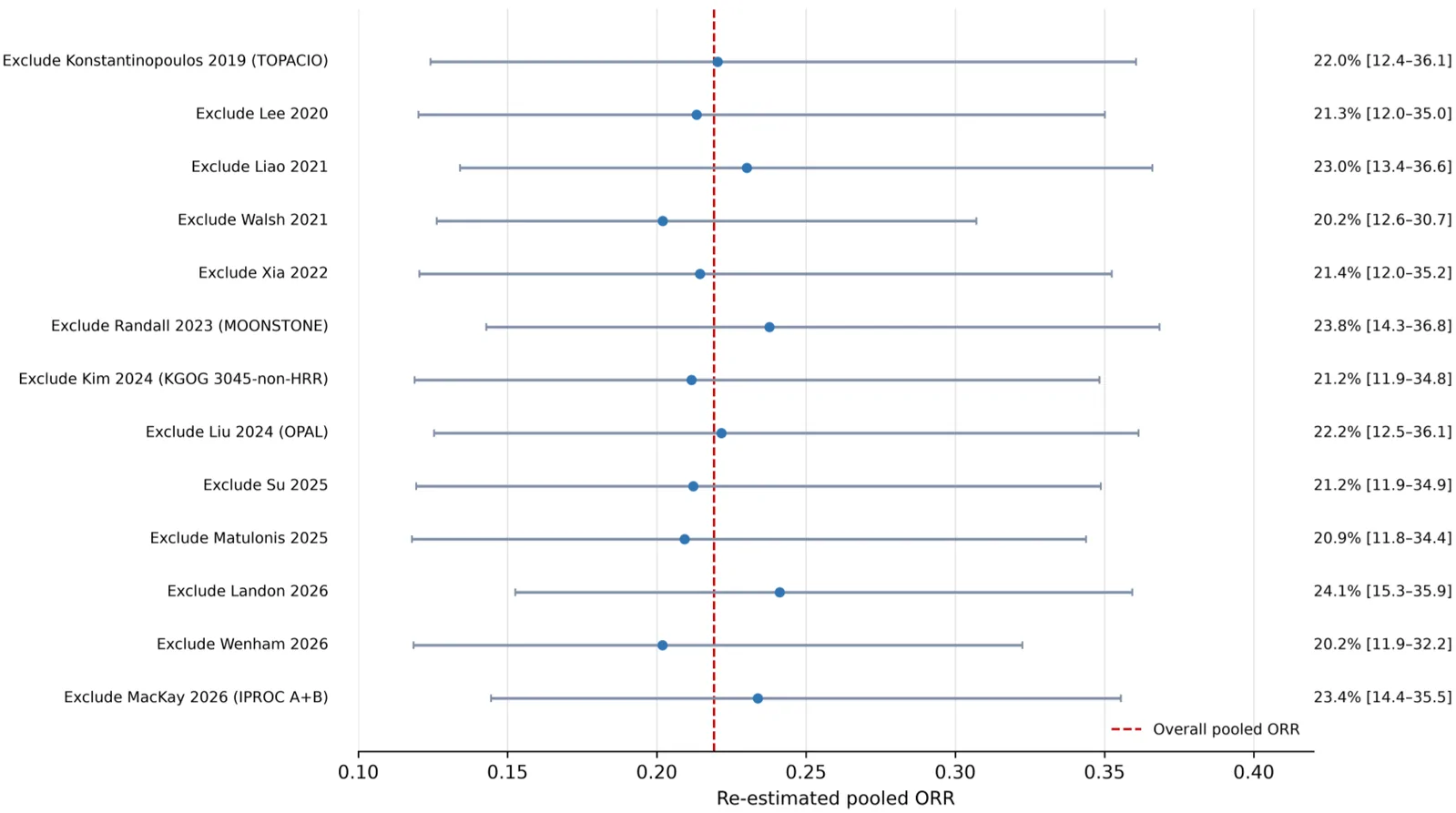
Leave-one-out sensitivity analysis of the primary objective response rate meta-analysis. The dashed line denotes the overall pooled estimate.

Leave-one-out estimates ranged from 20.2% to 24.1%, indicating that no single cohort fully explained the pooled ORR (**Figure 4**). Exploratory estimates were 32.6% (95% CI 13.0%–61.0%; I²=74.6%) for five chemotherapy-containing cohorts, 25.7% (95% CI 1.1%–91.8%; I²=0%) for two antiangiogenic cohorts, and 13.0% (95% CI 0.02%–99.0%; I²=56.9%) for two PARP-inhibitor cohorts. These very wide intervals, particularly in two-study subgroups, preclude reliable ranking of combination classes.

## 4 Discussion

This updated review provides a clearer estimate of what ICI-based combination therapy can and cannot achieve in platinum-resistant or platinum-refractory ovarian cancer. Across 13 prospective nonrandomized cohorts, the pooled objective response rate was 21.9%, and the pooled disease control rate across 10 compatible cohorts was 59.9%. The randomized evidence was more informative for clinical decision-making and did not support a uniform class effect: KEYNOTE-B96 demonstrated PFS and OS improvements with pembrolizumab plus weekly paclitaxel, whereas JAVELIN Ovarian 200, EORTC 1508, NRG-GY023, and AGO-OVAR 2.29 did not meet their principal efficacy objectives (8, 9, 32, 33, 36). These findings favor a regimen-specific interpretation rather than the proposition that adding any ICI to another active drug improves outcomes (39, 40). Single-arm activity and comparative efficacy require different interpretations. A response proportion describes outcomes within a treated cohort but does not establish the direction or magnitude of benefit relative to standard treatment. The present meta-analysis therefore synthesized compatible nonrandomized objective response and disease control data as proportions, whereas randomized trials retained their published hazard ratios and treatment contrasts. This design-stratified approach preserves the signal-generating role of phase II cohorts without conflating their estimates with comparative treatment effects.

The pooled response estimate indicates clinically observable activity, but its magnitude requires caution. Individual-study response estimates ranged from 0% in IPROC to 61.1% in the small cisplatin–gemcitabine–pembrolizumab cohort (1, 25). This dispersion was not explained by a single influential study in leave-one-out analysis. It probably reflects differences in treatment partner, prior bevacizumab or poly(ADP-ribose) polymerase inhibitor exposure, histology, biomarker enrichment, disease burden, and response-evaluable populations (41). Chemotherapy may enhance antigen release and immunogenic cell death; antiangiogenic treatment may reduce vascular endothelial growth factor-associated immune suppression; PARP inhibition may activate innate immune signaling; and antibody–drug conjugates may increase tumor-cell killing and antigen presentation (42). These mechanisms support testing specific combinations, but they do not establish therapeutic equivalence. The wide confidence intervals in the exploratory subgroups also preclude ranking chemotherapy, antiangiogenic, or PARP-inhibitor combinations (43, 44). The phase III results illustrate why biological rationale must be validated at regimen level. JAVELIN Ovarian 200 did not significantly improve PFS or OS with avelumab plus pegylated liposomal doxorubicin, and AGO-OVAR 2.29 did not show a significant survival benefit from adding atezolizumab to bevacizumab and nonplatinum chemotherapy (8, 36). In contrast, KEYNOTE-B96 showed PFS and OS benefit from pembrolizumab plus weekly paclitaxel (9). Differences in chemotherapy backbone, treatment schedule, immune context, prior therapy, PD-L1 distribution, and trial population may have contributed, but cross-trial comparisons cannot identify the responsible factor. The negative phase II trials are also informative: EORTC 1508 missed its PFS-at-6-month objective, both durvalumab-containing NRG-GY023 strategies crossed futility boundaries, and concurrent versus sequential durvalumab–tremelimumab produced similar immune-related PFS (29, 32, 33). KEYNOTE-B96 should therefore change the evidence base for its tested regimen and population, not retrospectively validate combinations that failed in other randomized studies.

Biomarker development remains an unresolved issue. PD-L1 expression, homologous recombination repair status, BRCA status, folate receptor-alpha expression, histologic subtype, and immune-microenvironment measures were not reported consistently enough for a predictive meta-analysis. Several studies deliberately enriched molecularly or antigen-defined populations, including the homologous recombination repair-selected KGOG 3045 cohorts and the folate receptor-alpha-directed mirvetuximab combination (1, 28, 30, 35). However, between-study associations cannot distinguish predictive effects from prognostic differences or regimen selection. Future trials should prespecify biomarker-positive and biomarker-negative analyses, use harmonized assays and cut points, retain an appropriate concurrent comparator, and report interaction tests. Participant-level data would also permit evaluation of continuous biomarkers and adjustment for prior treatment and clinical covariates. Safety should be interpreted by regimen rather than by checkpoint-inhibitor class. Trials variously reported treatment-emergent, treatment-related, immune-mediated, grade-specific, or selected adverse events over different observation windows. Pooling these constructs would produce a precise but clinically ambiguous estimate. In KEYNOTE-B96, grade 3 or worse treatment-related adverse events were more frequent with pembrolizumab than control, whereas AGO-OVAR 2.29 reported broadly similar proportions of grade 3 or worse adverse events between groups (9, 36). Pneumonitis was notable with mirvetuximab–pembrolizumab, while chemotherapy-containing regimens also produced expected hematologic and gastrointestinal toxicity (35). Standardized reporting should include treatment exposure, attribution, immune-mediated events, discontinuations, dose modifications, deaths, and late events so that benefit can be weighed against regimen-specific harm (40).

This review has several strengths. The eligibility criteria align the evidence with the defined clinical population. Report-level screening decisions and event counts are traceable in the supplementary tables. Randomized and nonrandomized designs are analyzed according to their inferential roles, RoB 2 and MINORS are applied separately, and zero-event handling and heterogeneity methods are stated explicitly. Leave-one-out analysis tests dependence on individual cohorts, while the decisions not to pool heterogeneous randomized hazard ratios or incompatible safety outcomes reduce false precision. Important limitations remain. The review was not prospectively registered. The evidence is based on aggregate data, preventing standardized reanalysis of response, survival, and biomarkers. Nonrandomized cohorts were small, some ended early, and selective publication of encouraging phase II signals cannot be excluded. Clinical heterogeneity remained substantial despite restricting platinum status; safety definitions and follow-up were inconsistent; and exploratory subgroups contained too few studies for reliable comparisons. Conventional tests for funnel-plot asymmetry would also be difficult to interpret for these heterogeneous proportions. For clinical practice, pembrolizumab plus weekly paclitaxel has randomized phase III evidence in the studied population, but that result should not be extrapolated to other ICI partners, maintenance settings, or platinum-sensitive disease. Future trials should use relevant controls, embedded translational sampling, prospective biomarker interaction testing, and common definitions for response and toxicity. They should also stratify prior bevacizumab, PARP-inhibitor, and immunotherapy exposure. The pooled proportions are best interpreted as descriptive benchmarks, whereas treatment decisions should be anchored to regimen-specific randomized evidence.

## 5 Conclusions

ICI-based combinations have heterogeneous activity in platinum-resistant/refractory recurrent ovarian cancer and should not be treated as a single effective class. Pembrolizumab plus weekly paclitaxel has phase III evidence of PFS and OS benefit, whereas several other randomized combinations were negative. Future work should validate specific regimens, harmonize safety and response definitions, and prospectively enrich for biologically plausible responders rather than extrapolating from pooled single-arm activity.

## Data Availability

The raw data supporting the conclusions of this article will be made available by the authors, without undue reservation.
