# Supplementary material for: Immune checkpoint inhibitor–based combination therapy for platinum-resistant or platinum-refractory recurrent ovarian cancer: a systematic review and meta-analysis of prospective trials
Source: Front Oncol. 2026 Aug 26;16:1837967. doi: 10.3389/fonc.2026.1837967 (PMC13553405; doi:10.3389/fonc.2026.1837967)
Supplement: Supplementary file 1 [file Table1.docx]

Supplementary Table S1. Search framework, and report-level exclusion log

| Source | Coverage/date | Search framework or method | Records |
| --- | --- | --- | --- |
| PubMed/MEDLINE, Embase, Web of Science, and CENTRAL | Inception to 23 July 2026 | Database-specific controlled vocabulary and free-text terms covering ovarian, fallopian tube, and primary peritoneal cancer; platinum resistance or refractoriness; PD-1/PD-L1 and CTLA-4 inhibitors and individual agents; combination treatment; and prospective phase II or III trials. Syntax was adapted to each database interface. | 674 |
| Citation searching | Reference lists and cited-by records of eligible trials/relevant reviews | Manual screening using the same PICOS criteria | 2 unique reports |

Report-level exclusion log

| Report | PMID | Primary exclusion reason |
| --- | --- | --- |
| Zsiros 2021 | 33211063 | Mixed platinum-sensitive/resistant population without extractable resistant-only data |
| Lampert 2020 | 32398324 | Predominantly, but not exclusively, platinum-resistant population |
| Liu 2019 | 31600397 | Mixed platinum-sensitive/resistant population without separable data |
| Taylor/LEAP-005 2024 | 38718741 | Mixed platinum-status cohort without separable resistant-only data |
| PESCO 2026 | 42129168 | Mixed platinum-status cohort without separable resistant-only data |
| BOLD 2024 | 38443333 | Relapsed population not restricted to platinum-resistant/refractory disease |
| Botensilimab/balstilimab phase 1 | 39910037 | Phase I dose-escalation study |
| Radiation plus checkpoint blockade | 40273550 | Phase I study |
| TRAMUNE | 34965951 | Phase Ib study |
| Atezolizumab plus bevacizumab phase Ib | 32723836 | Phase Ib study |
| ARRY-382 plus pembrolizumab | 35302585 | No extractable ovarian-specific efficacy cohort |
| AMBITION parent report | 35320892 | Overlapping population; later regimen-specific reports retained |
| JAVELIN cardiac safety report | 37665777 | Secondary safety analysis of an included trial |
| Azacitidine immune-correlate report 1 | 35838045 | Overlapping/secondary report |
| Azacitidine immune-correlate report 2 | 35671108 | Overlapping/secondary report |
| p53MVA/pembrolizumab immune-signature report | 38032111 | Secondary translational report |
| Pembrolizumab-lenvatinib institutional series | 40740417 | Retrospective study |
| ARTISTRY-7 | 39931671 | Protocol without peer-reviewed outcome results |

Supplementary Table S2. Detailed characteristics of included studies

| Report | PMID | Design | Eligible population | Regimen | N | Synthesis role |
| --- | --- | --- | --- | --- | --- | --- |
| Konstantinopoulos 2019 (TOPACIO) | 31194228 | Phase I/II, single-arm | Recurrent platinum-resistant ovarian carcinoma | Pembrolizumab + niraparib | 62 treated; 60 efficacy-evaluable | Primary ORR/DCR meta-analysis |
| Lee 2020 | 32771276 | Phase II, single-arm | Platinum-resistant ovarian cancer | Pembrolizumab + PLD | 26 treated; 23 response-evaluable | Primary ORR meta-analysis |
| Zamarin 2020 | 32503949 | Phase II, single-arm | Advanced platinum-resistant ovarian cancer | Durvalumab + TPIV200 FRα vaccine | 27 | Narrative synthesis; response unconfirmed |
| Liao 2021 | 34531249 | Phase II, single-arm | Recurrent platinum-resistant ovarian/FT/PP cancer | Pembrolizumab + low-dose carboplatin | 29 | Primary ORR/DCR meta-analysis |
| Walsh 2021 | 34081738 | Phase II, single-arm | Platinum-resistant ovarian cancer | Pembrolizumab + cisplatin + gemcitabine | 21 treated; 18 response-evaluable | Primary ORR/DCR meta-analysis |
| Pujade-Lauraine 2021 (JAVELIN Ovarian 200) | 34143970 | Phase III, open-label randomized | Platinum-resistant/refractory ovarian cancer | Avelumab + PLD vs avelumab vs PLD | 566 randomized; 188 combination arm | Randomized comparative synthesis |
| Xia 2022 | 35017154 | Phase II, single-arm ovarian cohort | Platinum-resistant recurrent ovarian cancer | Camrelizumab + famitinib | 37 | Primary ORR/DCR meta-analysis |
| Randall 2023 (MOONSTONE) | 37890345 | Phase II, single-arm | Recurrent platinum-resistant ovarian cancer | Dostarlimab + niraparib | 41 | Primary ORR/DCR meta-analysis |
| Kim 2023 (KGOG 3045-HRR) | 37602928 | Phase II, randomized noncomparative | HRR-mutated platinum-resistant ovarian cancer | Olaparib + durvalumab vs olaparib + cediranib | 30; 14 in ICI arm | Randomized evidence; not proportion-pooled |
| Hinchcliff 2024 | 38009662 | Phase II, randomized open-label | Recurrent platinum-resistant ovarian cancer | Concurrent vs sequential durvalumab + tremelimumab | 61 (23 concurrent; 38 sequential) | Randomized comparative synthesis |
| Kim 2024 (KGOG 3045-non-HRR) | 38246047 | Phase II umbrella trial | HRR-nonmutated platinum-resistant ovarian cancer | Durvalumab ± tremelimumab + chemotherapy | 58 across four ICI-containing arms | Primary ORR meta-analysis |
| Liu 2024 (OPAL) | 38754056 | Phase II, single-arm cohort | Pretreated platinum-resistant ovarian cancer | Dostarlimab + niraparib + bevacizumab | 41 | Primary ORR/DCR meta-analysis |
| Banerjee 2025 (EORTC 1508) | 40100121 | Phase II, randomized multicenter | Recurrent platinum-resistant ovarian cancer | Bevacizumab ± atezolizumab ± aspirin | 96 in three reported active cohorts | Randomized comparative synthesis |
| Lee 2025 (NRG-GY023) | 40192715 | Phase II, randomized selection design | Platinum-resistant ovarian cancer after bevacizumab | Durvalumab + cediranib ± olaparib vs SOC/other | 153; 86 in ICI-containing arms | Randomized comparative synthesis |
| Su 2025 | 40616958 | Phase II, single-arm | Platinum-resistant recurrent ovarian cancer | Toripalimab + low-dose lenvatinib | 33 | Primary ORR/DCR meta-analysis |
| Matulonis 2025 | 40700855 | Phase II, single-arm | FRα-positive platinum-resistant ovarian cancer | Pembrolizumab + mirvetuximab soravtansine | 56 treated; 55 response-evaluable | Primary ORR meta-analysis |
| Harter 2026 (AGO-OVAR 2.29) | 41337696 | Phase III, double-blind randomized | Recurrent ovarian cancer ineligible for platinum | Atezolizumab vs placebo + bevacizumab + chemotherapy | 574 | Randomized comparative synthesis |
| Landon 2026 | 41652014 | Phase II, nonrandomized | Platinum-resistant epithelial ovarian cancer | Pembrolizumab + oral azacitidine | 34 | Primary ORR/DCR meta-analysis |
| Wenham 2026 | 41558878 | Phase II, single-arm multicenter | Platinum-resistant epithelial ovarian cancer | Pembrolizumab + weekly paclitaxel | 42 enrolled; 41 ITT | Primary ORR/DCR meta-analysis |
| MacKay 2026 (IPROC A/B) | 42225005 | Phase II, nonrandomized platform | Platinum-resistant high-grade serous ovarian cancer | Durvalumab + mecbotamab or ozuriftamab vedotin | 20 (10 per substudy) | Primary ORR/DCR meta-analysis |
| Colombo 2026 (KEYNOTE-B96) | 41974150 | Phase III, double-blind randomized | Platinum-resistant recurrent ovarian cancer | Pembrolizumab vs placebo + weekly paclitaxel | 643 randomized (322 vs 321) | Randomized comparative synthesis |

Supplementary Table S3A. Extracted efficacy data and randomized comparative effects

Study-accounting summary: A total of 21 eligible prospective primary reports were included: 14 nonrandomized reports and 7 randomized trials. Among the 14 nonrandomized reports, 13 contributed compatible confirmed response data to the pooled ORR meta-analysis, whereas 1 report was retained for narrative synthesis only because its reported response was unconfirmed. The 7 randomized trials were summarized separately using trial-specific estimates and were not pooled with the nonrandomized reports.

| Report | PMID | Design and report-level handling | Confirmed ORR data | DCR data | Synthesis role |
| --- | --- | --- | --- | --- | --- |
| Konstantinopoulos 2019 (TOPACIO) | 31194228 | Phase I/II, single-arm | 11/60 (18.3%) | 39/60 (65.0%) | Pooled ORR and DCR analyses |
| Lee 2020 | 32771276 | Phase II, single-arm | 6/23 (26.1%) | Not pooled | Pooled ORR analysis |
| Zamarin 2020 | 32503949 | Phase II, single-arm | No confirmed response; 1 unconfirmed PR among 27 participants | Not pooled | Narrative synthesis only |
| Liao 2021 | 34531249 | Phase II, single-arm | 3/29 (10.3%) | 18/29 (62.1%) | Pooled ORR and DCR analyses |
| Walsh 2021 | 34081738 | Phase II, single-arm | 11/18 (61.1%) | 16/18 (88.9%) | Pooled ORR and DCR analyses |
| Xia 2022 | 35017154 | Phase II, single-arm ovarian cohort | 9/37 (24.3%) | 20/37 (54.1%) | Pooled ORR and DCR analyses |
| Randall 2023 (MOONSTONE) | 37890345 | Phase II, single-arm | 3/41 (7.3%) | 12/41 (29.3%) | Pooled ORR and DCR analyses |
| Kim 2024 (KGOG 3045-non-HRR) | 38246047 | Phase II umbrella trial; four eligible ICI-containing arms combined once at report level | 16/58 (27.6%) | Not pooled | Pooled ORR analysis |
| Liu 2024 (OPAL) | 38754056 | Phase II, single-arm cohort | 7/41 (17.1%) | 30/41 (73.2%) | Pooled ORR and DCR analyses |
| Su 2025 | 40616958 | Phase II, single-arm | 9/33 (27.3%) | 18/33 (54.5%) | Pooled ORR and DCR analyses |
| Matulonis 2025 | 40700855 | Phase II, single-arm | 17/55 (30.9%) | Not pooled | Pooled ORR analysis |
| Landon 2026 | 41652014 | Phase II, nonrandomized | 1/34 (2.9%) | 17/34 (50.0%) | Pooled ORR and DCR analyses |
| Wenham 2026 | 41558878 | Phase II, single-arm multicenter | 19/41 (46.3%) | 32/41 (78.0%) | Pooled ORR and DCR analyses |
| MacKay 2026 (IPROC A/B) | 42225005 | Phase II nonrandomized platform report; two eligible substudies combined once at report level | 0/20 (0.0%) | 9/20 (45.0%) | Pooled ORR and DCR analyses |

Supplementary Table S3B. Evidence from the seven randomized trials

| Trial/report | PMID | Design or contrast | Reported efficacy estimate | Interpretation and synthesis role |
| --- | --- | --- | --- | --- |
| Pujade-Lauraine 2021 (JAVELIN Ovarian 200) | 34143970 | Phase III randomized trial; avelumab + PLD versus PLD | ICI-combination-arm ORR: 25/188 (13.3%); PFS: HR, 0.78 (93.1% CI, 0.59–1.24); OS: HR, 0.89 (88.85% CI, 0.74–1.24) | Primary PFS and OS endpoints were not met; trial-specific estimates reported separately |
| Kim 2023 (KGOG 3045-HRR) | 37602928 | Randomized, two-arm, explicitly noncomparative phase II trial; olaparib + durvalumab versus olaparib + cediranib | ORR in the ICI-containing arm: 6/14 (42.9%); no formal comparative effect estimate reported | Retained as randomized evidence but not interpreted as a between-arm treatment effect |
| Hinchcliff 2024 | 38009662 | Randomized phase II trial; concurrent versus sequential durvalumab/tremelimumab | Concurrent-arm ORR: 2/23 (8.7%); median immune-related PFS: 1.87 versus 1.84 months; P = .402; OS not reported | No comparative efficacy signal; trial-specific results reported separately |
| Banerjee 2025 (EORTC 1508) | 40100121 | Randomized multicenter phase II trial; bevacizumab + atezolizumab with or without aspirin versus bevacizumab | ORR: 19% and 15% in the two ICI-containing arms; PFS at 6 months: 8/32 and 8/32 versus 7/32; OS not reported in the abstract | Primary PFS-at-6-month objective was not reached in any arm |
| Lee 2025 (NRG-GY023) | 40192715 | Randomized phase II selection trial; durvalumab + cediranib with or without olaparib versus SOC | Combined ORR in the ICI-containing arms: 12/86 (14.0%); PFS: HR, 1.108 (95% CI, 0.63–1.96) and HR, 1.003 (95% CI, 0.56–1.80), respectively | Both ICI-containing arms crossed the prespecified futility boundaries |
| Harter 2026 (AGO-OVAR 2.29) | 41337696 | Phase III double-blind randomized trial; atezolizumab + bevacizumab + chemotherapy versus placebo + bevacizumab + chemotherapy | PFS: HR, 0.87 (95% CI, 0.73–1.04; P = .12); OS: HR, 0.83 (95% CI, 0.68–1.01; P = .06) | No statistically significant PFS or OS benefit |
| Colombo 2026 (KEYNOTE-B96) | 41974150 | Phase III double-blind randomized trial; pembrolizumab + weekly paclitaxel versus placebo + weekly paclitaxel | PFS: HR, 0.70 (95% CI, 0.58–0.84); OS: HR, 0.82 (95% CI, 0.69–0.97) | Statistically significant PFS and OS benefit in the intention-to-treat population |

Each unique eligible primary publication was counted as one report. Trial arms and cohorts were not counted as separate reports. When a nonrandomized report contained multiple eligible ICI-containing cohorts or substudies, these were combined once at the report level for the primary proportion meta-analysis. ORR was defined as confirmed complete response plus partial response. The unconfirmed partial response reported by Zamarin et al. was not counted as a confirmed objective response; this report was therefore retained for narrative synthesis only. DCR was pooled only when conventional complete response plus partial response plus stable disease counts and a compatible denominator were available. For IPROC A/B, nine participants had stable disease and no objective response; the combined DCR was therefore 9/20. All efficacy data were extracted from the primary publications and associated full reports or supplementary materials where available.

Abbreviations: Bev, bevacizumab; CI, confidence interval; DCR, disease control rate; HR, hazard ratio; ICI, immune checkpoint inhibitor; ORR, objective response rate; OS, overall survival; PFS, progression-free survival; PLD, pegylated liposomal doxorubicin; PR, partial response; SOC, standard of care.

Supplementary Table S4. Design-appropriate risk-of-bias assessment

Part A. Randomized trials: Cochrane RoB 2 judgments

| Trial | Randomization process | Deviations from intended interventions | Missing outcome data | Outcome measurement | Selection of the reported result | Overall |
| --- | --- | --- | --- | --- | --- | --- |
| JAVELIN Ovarian 200 | Low | Some concerns | Low | Low | Low | Some concerns |
| KGOG 3045-HRR | Some concerns | Low | Low | Low | Low | Some concerns |
| Hinchcliff 2024 | Some concerns | Some concerns | Low | Low | Low | Some concerns |
| EORTC 1508 | Low | Some concerns | Low | Low | Low | Some concerns |
| NRG-GY023 | Some concerns | Some concerns | Low | Low | Low | Some concerns |
| AGO-OVAR 2.29 | Low | Low | Low | Low | Low | Low |
| KEYNOTE-B96 | Low | Low | Low | Low | Low | Low |

Part B. Nonrandomized trials: MINORS (maximum 16 for noncomparative studies)

| Study | MINORS score (/16) | Principal limitation |
| --- | --- | --- |
| TOPACIO | 13 | Prospective phase II efficacy cohort; no concurrent comparator |
| Lee 2020 | 13 | Prospective single-arm study; small response-evaluable cohort |
| Zamarin 2020 | 12 | Unconfirmed response and small single-arm cohort |
| Liao 2021 | 13 | Prospective single-arm study; limited sample size |
| Walsh 2021 | 12 | Early closure and 18 response-evaluable participants |
| Xia 2022 | 13 | Ovarian cohort of a basket study |
| MOONSTONE | 13 | Prospective; terminated for futility |
| KGOG 3045-non-HRR | 13 | Umbrella arms without a common randomized ICI comparator |
| OPAL | 13 | Prospective single-arm cohort |
| Su 2025 | 12 | Preliminary phase II report |
| Matulonis 2025 | 13 | Biomarker-selected, nonrandomized cohort |
| Landon 2026 | 12 | Small nonrandomized translational trial |
| Wenham 2026 | 13 | Prospective multicenter single-arm study |
| IPROC A/B | 12 | Two Simon two-stage cohorts stopped after stage 1 |

RoB 2 and MINORS judgments were made at report level for the outcomes used in this review. A MINORS score was used descriptively and was not converted into a binary low/high-risk threshold.
